# Genome assembly, annotation, and comparative analysis of the cattail *Typha latifolia*

**DOI:** 10.1093/g3journal/jkab401

**Published:** 2021-11-22

**Authors:** Shane D Widanagama, Joanna R Freeland, Xinwei Xu, Aaron B A Shafer

**Affiliations:** 1 Department of Computer Science, Trent University, Peterborough, ON K9L 0G2, Canada; 2 Department of Biology, Trent University, Peterborough, ON K9L 0G2, Canada; 3 Department of Ecology, College of Life Sciences, Wuhan University, Wuhan 430072, China; 4 Department of Forensic Sciences, Trent University, Peterborough, ON K9L 0G2, Canada

**Keywords:** PacBio long-read sequencing, illumina short-read, *de novo*, Typhaceae, bulrush, broadleaf cattail, hybrids

## Abstract

Cattails (*Typha* species) comprise a genus of emergent wetland plants with a global distribution. *Typha latifolia and Typha angustifolia* are two of the most widespread species, and in areas of sympatry can interbreed to produce the hybrid *Typha* × *glauca*. In some regions, the relatively high fitness of *Typha* × *glauca* allows it to outcompete and displace both parent species, while simultaneously reducing plant and invertebrate biodiversity, and modifying nutrient and water cycling. We generated a high-quality whole-genome assembly of *T. latifolia* using PacBio long-read and high coverage Illumina sequences that will facilitate evolutionary and ecological studies in this hybrid zone. Genome size was 287 Mb and consisted of 1158 scaffolds, with an N50 of 8.71 Mb; 43.84% of the genome were identified as repetitive elements. The assembly has a BUSCO score of 96.03%, and 27,432 genes and 2700 RNA sequences were putatively identified. Comparative analysis detected over 9000 shared orthologs with related taxa and phylogenomic analysis supporting *T. latifolia* as a divergent lineage within Poales. This high-quality scaffold-level reference genome will provide a useful resource for future population genomic analyses and improve our understanding of *Typha* hybrid dynamics.

## Introduction

Cattails (*Typha* spp.) are aquatic macrophytes that are essential components of wetlands around the world (reviewed in Bansal *et al.* 2019). These macrophytes often dominate ecosystems through a combination of rapid growth, large size, and sexual and asexual reproduction (Yeo 1964; Andrews and Pratt 1978; Miller and Fujii 2010). Cattails are vital to many wetlands where they cycle nutrients, provide habitat, and aid in bioremediation (Grosshans 2014; Svedarsky *et al.* 2016; Bonanno and Cirelli 2017). However, cattails can also dominate wetlands and in recent decades invasive cattails have been identified in numerous regions, often following anthropogenic changes that have altered water cycles and increased nutrient loads in wetlands (reviewed in Zedler and Kercher 2004; Bansal *et al.* 2019). Invasive cattails often form monotypic stands with deleterious effects on local plants and animals, wetland water cycling, and biogeochemical cycles (Yeo 1964; Grace and Harrison 1986; Farrer and Goldberg 2009; Lawrence *et al.* 2016).

*Typha latifolia* is likely the most widespread cattail species, occurring on every continent except Antarctica (Smith 1987). *Typha angustifolia* is also widespread throughout the temperate northern hemisphere (Grace and Harrison 1986), including North America where it was likely introduced from Europe several centuries ago (Ciotir *et al.* 2013, 2017). In some regions of sympatry *T. latifolia* and *T. angustifolia* interbreed to produce the hybrid *Typha* × *glauca* (Grace and Harrison 1986; Ciotir *et al.* 2017; Bansal *et al.* 2019). In regions surrounding, the Laurentian Great Lakes and St. Lawrence Seaway in North America, *Typha* × *glauca* exhibits heterosis (Bunbury-Blanchette *et al.* 2015; Zapfe and Freeland 2015), and is more abundant than its parental species (Kirk *et al.* 2011; Freeland *et al.* 2013; Pieper *et al.* 2020). In addition to displacing both parental species, invasive *Typha* × *glauca* reduces native plant and invertebrate biodiversity (Tuchman *et al.* 2009; Lawrence *et al.* 2016), and alters nutrient cycling and community structure in wetlands (Tuchman *et al.* 2009; Larkin *et al.* 2012; Geddes *et al.* 2014; Lishawa *et al.* 2014; Lawrence *et al.* 2017).

Although dominant in some regions of North America, *Typha* × *glauca* is uncommon in other regions where the parental species are sympatric, including Europe (Ciotir *et al.* 2017), eastern Canada (Freeland *et al.* 2013), and China (Zhou *et al.* 2016). It is not well understood why hybrids are dominant in some regions but not others, although Tisshaw *et al.* (2020) suggested that hybrids may be limited in coastal wetlands because they have difficulty germinating in salt-rich environments. In addition, although advanced-generation and back-crossed hybrids have been experimentally generated (Pieper *et al.* 2017) and identified in natural populations (*e.g.*, Kirk *et al.* 2011; Freeland *et al.* 2013; Pieper *et al.* 2020), it has not been possible to differentiate advanced-generation and backcrossed hybrids based on the small number of species-specific molecular markers that are currently available (Snow *et al.* 2010; Kirk *et al.* 2011). Morphology-based assessments are also unreliable due to overlapping phenotypes (Tangen *et al.* 2021).

A genome-wide suite of SNPs specific to one or the other parent species would greatly facilitate investigations into the dynamics of the *Typha* × *glauca* hybrid zone, which is now expanding westwards from the Great Lakes region into the Prairie Pothole Region of Canada and the USA (Tangen *et al.* 2021). Genome characterization would also facilitate investigations into local adaptation, introgression, and hybrid dynamics; this in turn may inform future management of *T. latifolia*, which is predicted to experience a dramatically reduced distribution following climate change (Xu *et al.* 2013), likely to the detriment of wetlands throughout its widespread native distribution. Here, we report the genome assembly, annotation, and analysis of *T. latifolia*, the first fully sequenced species in the family Typhaceae.

## Materials and methods

### Sampling and sequencing

**Figure 1:**
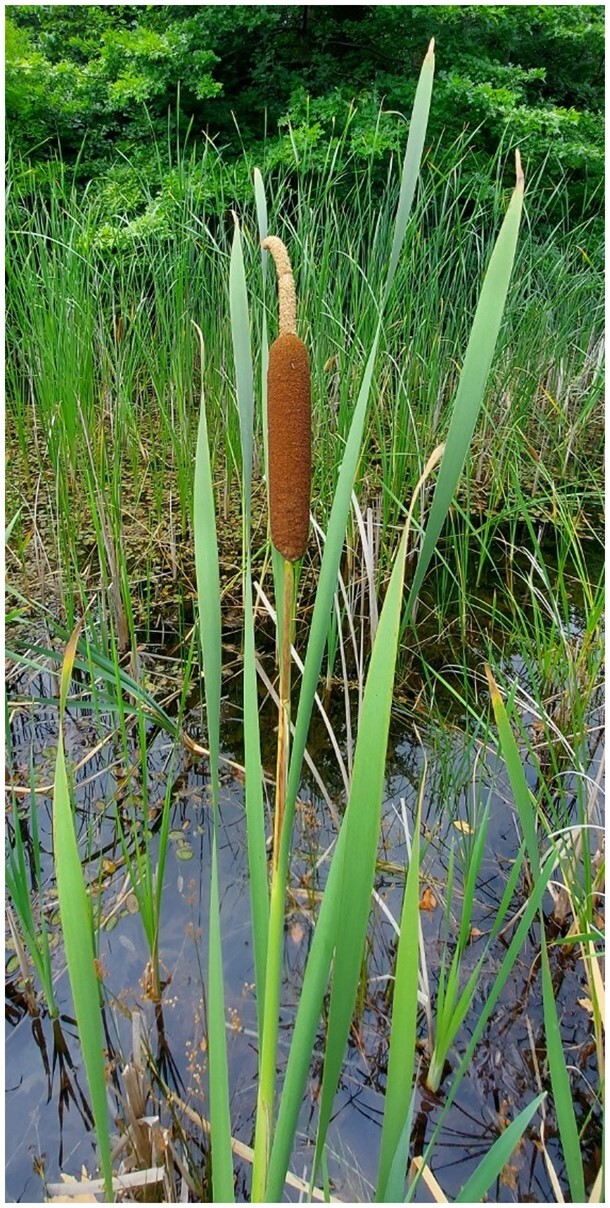
Broadleaf cattail (*Typha latifolia*). Photo by Joanna Freeland.

Leaves of a known *T. latifolia* plant (Figure  1) were taken from an individual grown at Trent University’s (ON, Canada) greenhouse and ground in liquid nitrogen. DNA was immediately extracted using an E.Z.N.A Plant DNA Kit (Omega Bio-Tek, Inc. GA, USA) following the manufacturer’s instructions for frozen material and eluted in a final volume of 100 μl. DNA quality was assessed on a Tapestation (Agilent Technologies, CA, USA) before being sent to The Centre for Applied Genomics, Toronto, ON, for sequencing. Paired-end reads were sequenced on a single lane of an Illumina HiSeqX system (Illumina, Inc. CA, USA). Long-reads (LR) were sequenced on one SMRT HiFi cell using a PacBio Sequel II system (Pacific Biosciences of California, Inc. CA, USA).

### Genome assembly

Adapters and low-quality bases were trimmed from paired-end short reads (SRs) using Trimmomatic -v0.36 (Bolger *et al.* 2014). The optimal k-mer and genome size from the SRs were estimated using Kmergenie -v1.7051 (Chikhi and Medvedev 2014). We then applied a four-step hybrid assembly: (1) SR assemblies were generated using multiple pipelines: ABySS –v2.2.4 (Jackman *et al.* 2017) with default settings, 32 threads, a 125 GB total memory; SOAPdenovo2 -r240 (Luo *et al.* 2012) using default settings, 16 threads, 400 GB of total memory; and Platanus -v1.2.4 (Kajitani *et al.* 2014) using default settings, 32 threads, a 343 GB memory limit, a total memory of 375 GB. We also downsampled the SR data to 20% because really high coverage can be problematic for de Bruijn graphs (Richards and Murali 2015). The SR assembly with highest quality and completeness was selected by calculating contig N50, total contig length as a percentage of estimated genome size, and the number and percentage of contigs longer than 50 Kb. (2) SR contigs were then aligned to raw LR to create scaffolds using DBG2OLC -v20160205 (options: k 17, AdaptiveTh 0.001, KmerCovTh 2, MinOverlap 20, RemoveChimera 1) (Ye *et al.* 2016). (3) LR contigs were assembled using Canu –v2.0 (Koren *et al.* 2017) using the expected genome size inferred from Kmergenie, a maximum memory of 124 GB, and a maximum of 32 threads. And (4) the hybrid assembly (Step 2) was merged with Canu LR contigs (Step 3) using QuickMerge –v0.3.0 (Chakraborty *et al.* 2016) following the 2-step strategy described by Solares *et al.* (2018). Pilon –v1.23.0 (Walker *et al.* 2014) was used to polish the genome by mapping the Illumina SR back to the genome, thereby correcting base errors and small misassemblies. The final assembly was then submitted and assessed for sequencing and assembly artifacts by NCBI.

### Genome evaluation and annotation

The *T. latifolia* hybrid genome was assessed for completeness using Benchmarking Universal Single-copy Orthologs (BUSCO) –v3.0.2 (Simão *et al.* 2015). BUSCOs of the lineage Liliopsida were assessed from OrthoDB release 10 (Waterhouse *et al.* 2013). We aligned available chloroplast and mitochondrial genomes (Supplementary Table S1) to our assembly using NUCmer -v3.23 (Kurtz *et al.* 2004) to identify plastid genomes; scaffolds with long matches (>5000 bp) were extracted and further validated on NCBI Blast. Structural and functional annotation was done in the GenSAS -v6.0 annotation pipeline (Humann *et al.* 2019). We masked interspersed and simple repetitive elements throughout the genome using a database developed through repeat modeler -v2.0.1 (Smit and Hubley 2008–2015) in conjunction with repeat masker -v4.0.7 with the NCBI/rmblast search engine, and quick sensitivity (Smit *et al.* 2013–2015). The hardmasked version of the genome sequence was used for feature prediction. Available RNA-seq data from *T. angustifolia* (SRR15541138) were mapped to the genome using hisat2 -v2.1.0 (Kim *et al.* 2019) and gene prediction with the RNA-seq alignments was performed by the BRAKER2 -v2.1.1 pipeline, which uses Augustus and GeneMark-ET (Lomsadze *et al.* 2014; Brůna *et al.* 2021). ncRNA was predicted using tRNAscan-SE -v2.0 (Lowe and Eddy 1997) and Infernal -v1.1.3 (Nawrocki and Eddy 2013). Refinement of the official gene set was performed with an available *T. latifolia* transcriptome (Moscou 2017) using PASA -v2.3.3 (Haas *et al.* 2003). Last, our assembly (N50 scaffolds) was aligned and compared to a recent *T. latifolia* hybrid scaffold assembly (JAAWWQ010000000) using Mummer4 (Marçais *et al.* 2018) requiring a minimum alignment (*l*) of 10,000 bp.

### Comparative genomics

Single-copy orthologous genes from five other species were identified using OrthoVenn2 with an *e*-value cutoff of 1e-5, an inflation value of 1.5, and the annotation, protein similarity network, and cluster relationship network enabled (Xu *et al.* 2019). OrthoVenn2 performs all-against-all genome-wide protein comparisons and groups genes into clusters with the Markov Clustering Algorithm, where a cluster is made up of orthologs and paralogs (Wang *et al.* 2015). Here we selected *Oryza sativa Japonica, Brachypodium distachyon*, and *Sorghum bicolor* from the family Poaceae, and *Ananas comosus* from the family Bromeliaceae*. Arabidopsis thaliana* from the Brassicaceae family was chosen as the dicot outgroup. All proteomes were from the Ensemble database release 104 (Howe *et al.* 2021). The identified orthologous sequences were then aligned using MAFFT -v7.741 (Katoh and Standley 2013) and concatenated into single sequences by species using SeqKit -v0.15.0 (Shen *et al.* 2016). Maximum likelihood phylogenetic analysis was performed using RAxML -v8.2.12 (Stamatakis 2014) with the PROTGAMMAAUTO substitution model (Darriba *et al*. 2012). The phylogenetic tree divergence times were estimated using MCMCTree from the PAML package -v4.9j (Yang 1997, 2007), and was calibrated using the divergence time between *Sorghum* and *Oryza* (42–52 Mya) (Kumar *et al.* 2017).

## Results

### Sequencing data and genome assembly

A total of 138.6 Gb of raw 151 bp Illumina reads were sequenced (Supplementary Table S2). Quality filtering and trimming removed 6.5 Gb of sequence data. A recommended k-mer size of 101 bp and estimated genome size of 257 Mb was estimated from the short-read data (Supplementary Figure S1). LR data from the Pacbio Sequel II generated 86.8 Gb of raw data: this included 7,244,218 subreads with a mean length of 11,978.2 bp. All sequencing reads were deposited in the NCBI Sequence Read Archive (Accession No PRJNA751759). The ABySS assembly that used 100% of Illumina reads had the most contiguous genome and was used for the assembly (Supplementary Table S3). This AbySS SR assembly had a N50 of 0.011 Mb, 365,565 contigs, and 362 contigs longer than 50 Kb (Supplementary Table S3). The DBG2OLC assemblies (ABySS contigs + raw long reads) improved the assembly statistics but resulted in a smaller than expected genome (Table  1); here we calculated N50 of 0.132 Mb, 1840 contigs, and 1445 contigs longer than 50 Kb (Supplementary Table S4). The LR Canu assembly had an N50 of 8.706 Mb, and contained 1189 contigs, 821 of which were longer than 50 Kb (95.54%) (Table  1). The final merged and polished hybrid genome assembly—DGB2OLC assembly 1 + Canu—was 286.7 Mb with plastids removed. We estimated an N50 of 8.71 Mb, and consisted of 1158 scaffolds (Table  1). Step 4 resulted in the merger of three scaffolds and the subsequent removal of 30 short scaffolds by NCBI vetting. The GC content was 38.05% (Table  1). The polished genome has been deposited in the NCBI genome database (JAIOKV000000000). We observed a high degree of synteny between our alignment and similar hybrid assembly of *T. latifolia* (Supplementary Figure S2), although mapping success of unrelated individuals was significantly higher in our genome (Supplementary Figure S3).

**Table 1:** Genome assembly statistics of our four-step hybrid assembly

	SR contig assembly—step 1	DB2OLC contig assembly—step 2	LR contig assembly—step 3	Merged polished scaffolds—step 4
Genome size (Mb)	263.74	193.16	287.63	286.77
Contigs/scaffolds	365,565	1,840	1,190	1,158
N50/L50	11.43 Kb/5,314	132.07 Kb/412	8.71 Mb/13	8.71 Mb/13
N90/L90	201 bp/193,744	52.95 Kb/1,358	58.92 Kb/530	58.94 Kb/523
Max sequence length	154.73 Kb	934.40 Kb	18.70 Mb	18.70 Mb
Scaffolds > 10 Kb	6,048	1,833	1,140	1,132
Scaffolds > 25 Kb	1,759	1,785	1,127	1,120
Scaffolds > 50 Kb	362	1,445	821	816
% of scaffolds > 50 Kb	9.46	92.34	95.54	95.59
GC content (%)	38.40	38.50	38.11	38.05

Short read (SR) contig assembly consists of contigs assembled from 100% of the Illumina reads using the ABySS assembler. The DBG2OLC assembler combined the 100% ABySS contigs and PacBio long reads. The long read (LR) contig assembly was generated from only PacBio long reads using Canu. The merged polished scaffolds are from merging the DBG2OLC assembly and LR contigs and were polished using Pilon.

### Assembly quality and annotation

**Figure 2:**
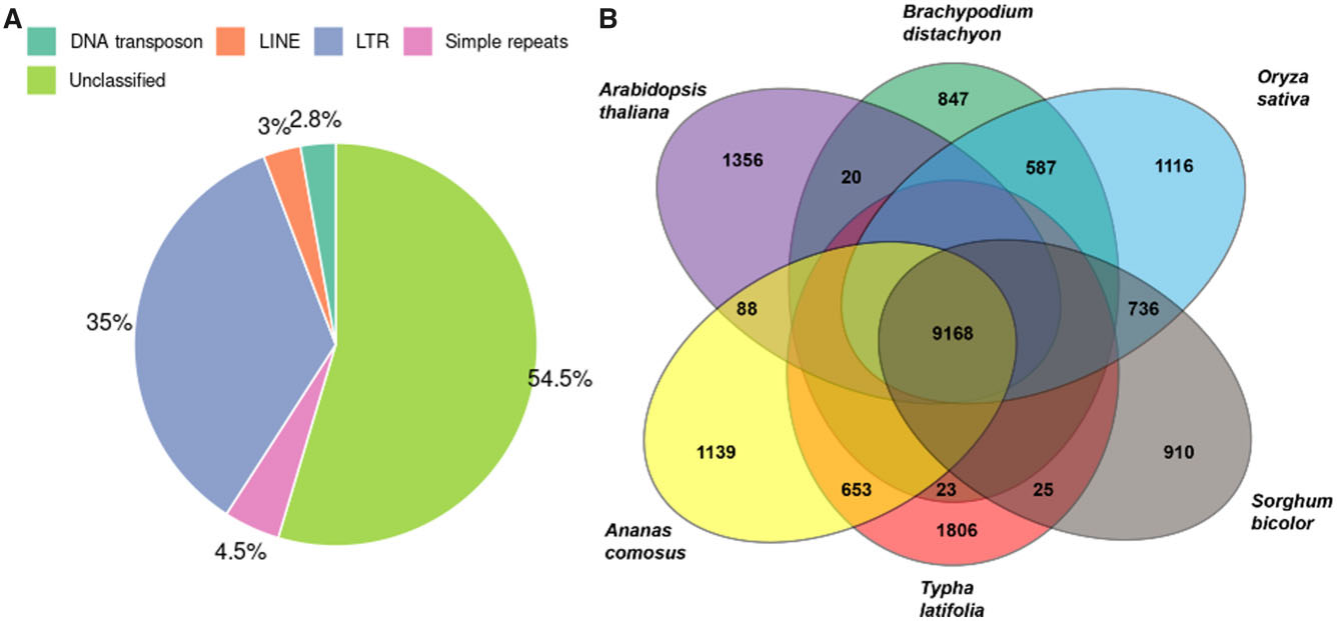
(A) Percentages of repeat types masked in the *T. latifolia* genome. Types of repeats include DNA transposons, long interspersed nuclear elements (LINEs), long terminal repeats (LTRs), unclassified repeats, and simple repeats. (B) Venn diagram of orthologous gene clusters among the broadleaf cattail (*Typha latifolia*), pineapple (*Ananas comosus*), thale cress (*Arabidopsis thaliana*), stiff brome (*Brachypodium distachyon*), rice (*Oryza sativa Japonica*), and broom-corn (*Sorghum bicolor*). Only the numbers of ortholog clusters of adjacent species, those common to all species, and those unique to each species are labeled.

Approximately 96.03% of BUSCOs were identified (3148 of 3278) in the assembly. Of these, 3025 BUSCOs were complete (92.28%), and 123 were fragmented. Of the complete BUSCOs, 2461 were single-copies, and 564 had duplicates. Repeats represented 43.84% of the genome (see breakdown in Figure  2). The chloroplast genome was detected and split on two scaffolds, while the entire mitochondrial genome appeared to be assembled (Supplementary Table S5). Total repeat content of the genome is consistent with other Poales genomes (Kawahara *et al.* 2013; Ming *et al.* 2015; Redwan *et al.* 2016): long interspersed nuclear elements (LINEs) comprised 1.22% of the genome, while short interspersed nuclear elements (SINEs) were not detected in the genome (Table  2). The annotation pipeline produced 27,432 genes, which coded for 34,911 proteins and 34,974 mRNA sequences. 2095 rRNA, 502 tRNA, and 214 miRNA sequences were putatively identified, which are similar to sequence counts in related plants (Supplementary Table S6). No snRNA were identified.

**Table 2:** Summary of repeats masked in the *Typha latifolia* genome

	Length (bp)	Percentage of genome (%)
SINE	0	0
LINE	3,499,426	1.22
LTR elements	44,172,983	15.35
DNA elements	3,735,882	1.30
Unclassified	68,736,281	23.88
Small RNA	0	0
Satellites	0	0
Simple repeats	5,689,134	1.98
Low complexity	670,871	0.23
Total	126,178,695	43.84

### Comparative genomic analyses

**Figure 3:**
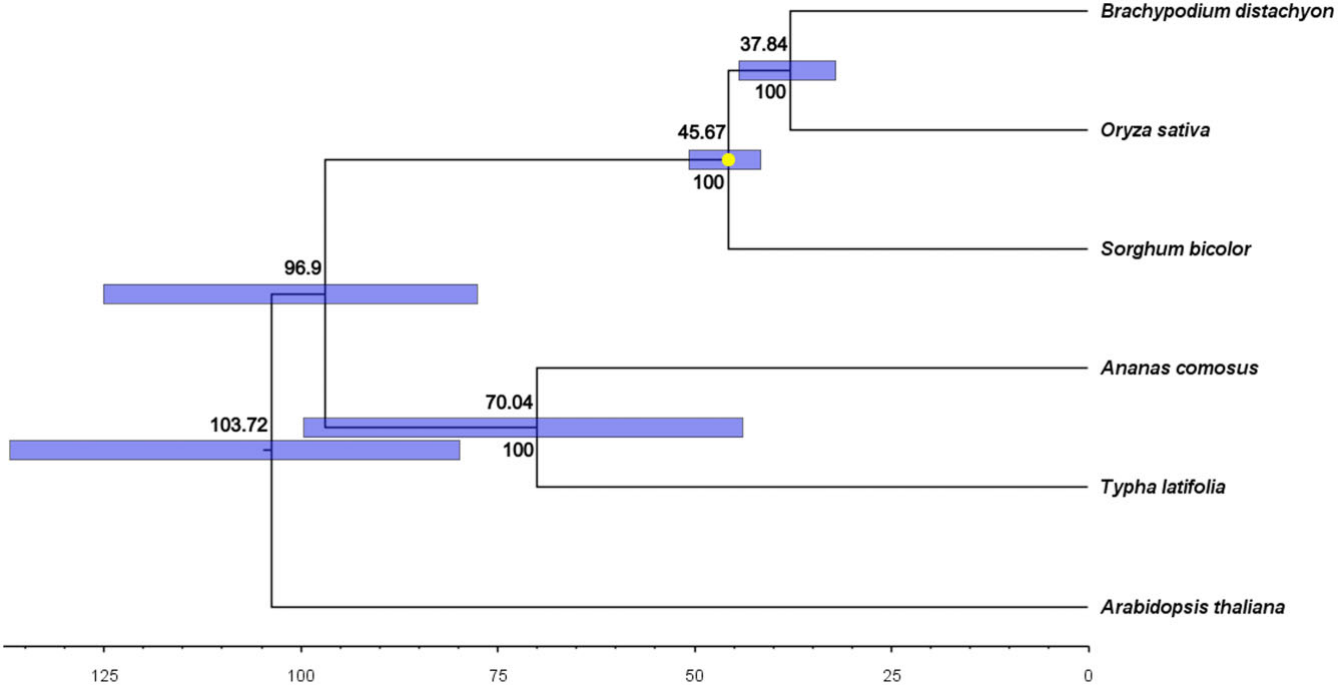
Phylogenetic tree with divergence times, based on the alignment of 1900 single-copy gene clusters. Ninety-five percent credible divergence times are shown as blue bars and were estimated using MCMCTree. Divergence times and bootstrap values are shown above and below the nodes, respectively. The yellow dot indicates the calibration point.

Ortholog clustering analysis revealed 9168 gene families were shared among *T. latifolia, A. comosus, A. thaliana, B. distachyon, O. sativa*, and *S. bicolor* (Figure  2). In total, 1806 gene families were found to be unique to *T.latifolia* (Figure  2). We aligned 1900 single-copy gene clusters to conduct phylogenomic analysis (Figure  3). The phylogenomic tree generated supports a divergent *Typha* position in Poales (Figure  3), with *Typha* forming a separate clade with pineapples (*A.comosus*). We estimated the bromeliad lineage (*Typha-Ananas*) to be approximately 70 million years old.

## Discussion

The Typhaceae family (order Poales) is diverse, comprising over 50 recognized species (Christenhusz and Byng 2016). Species in this family are essential components of marshes and wetlands around the world (reviewed in Bansal *et al.* 2019), and play a role in both bioremediation and the production of biofuel material (Grosshans 2014; Svedarsky *et al.* 2016; Bonanno and Cirelli 2017). This is the first published whole-genome assembly from a member of the Typhaceae family and adds to the previously characterized chloroplast genome sequences of *T.latifolia, T. orientalis*, and *Sparganium stoloniferum* (Guisinger *et al.* 2010; Su *et al.* 2019; Liu *et al.* 2020). The assembly and annotation of the Cattail genome, which we note was a key recommendation from a diverse team of *Typha* researchers (Bansal *et al.* 2019), will likely be an important tool in wetland management.

We explored a variety of SR assemblers given the varying quality of outputs seen in Assemblathon metrics (Bradnam *et al.* 2013). Of the three SR assemblers used, ABySS produced longer, fewer contigs and scaffolds, which is consistent with previous comparisons (Bradnam *et al.* 2013). ABySS’ de Bruijn approach did not have issues with the high coverage Illumina data (*e.g.*, Richards and Murali 2015). We combined with SR assembly with raw long read data using DGB2OLC (Ye *et al.* 2016) as previous plant genomes with similar data showed promising assembly statistics (*i.e*., Daccord *et al.* 2017; Hatakeyama *et al.* 2018; Zhou *et al.* 2019). The long reads in the DBG2OLC assembly captured more lengthy and repetitive regions, resulting in a sharp rise in contig N50 and contigs 50 Kb or longer (Table  1). However, the backbone of the final assembly was the PacBio Canu assembly (Table  1 and Supplementary Table S4), with the benefit of this strategy being the removal of chimeras with polished assemblies having very low error rates (Ye *et al.* 2016).

Accurate genomes are important as sequencing errors can affect both nucleotide diversity and the discovery of markers (Clark and Whittam 1992). LR sequencing further facilitates detection of structural variants (Amarasinghe *et al.* 2020), which might be useful in studying *Typha* dynamics given the link to hybridization and speciation (Weissensteiner *et al.* 2020). The final assembly consists of only 1158 scaffolds, with ~95% being 50 Kb or longer; *T. latifolia* possessed higher N50 and fewer scaffolds compared to other plant genome assemblies that used PacBio sequencing and similar hybrid assembly pipelines (Redwan *et al.* 2016; Hatakeyama *et al.* 2018; Reuscher *et al.* 2018). With 96.03% of BUSCOs detected and 92.28% of them complete in our assembly, this would suggest a high-quality annotation. The topology of the phylogenomic tree that we reconstructed based on whole-genome sequences of six species agrees with previously inferred evolutionary relationships (*e.g.*, Darshetkar *et al.* 2019) by grouping together the two bromeliad species (*T. latifolia* and *A. comosus*) and the three graminid species (*O. sativa, S. bicolor*, and *B. distachyon*), and by inferring a more recent divergence date for graminids compared to bromeliads. Our estimated *Typha* lineage age of approximately 70 million years exceeds an earlier estimate of between 22.64 and 57.60 mya that was based on seven cpDNA markers (Zhou *et al.* 2018), although is comparable to the estimate of 69.5 Myr that was based on a combination of fossil records and cpDNA sequences (Bremer 2000). We acknowledge that the use of whole-genome sequences to infer divergence times is still in its infancy, and caution that factors such as rate heterogeneity may complicate such inferences (reviewed in Smith *et al.* 2018).

The *T. latifolia* reference genome now allows for studying *Typha* and hybridization at the molecular level, for example, by providing insight into the adaptation of *T. latifolia* populations that may be threatened by climate change, and facilitating research into potentially important processes such as hybrid breakdown in invasive *Typha* hybrids. In addition, genome-wide markers could provide insight into why the hybrid *Typha* × *glauca* is dominant in some areas (Kirk *et al.* 2011; Freeland *et al.* 2013) but uncommon in others (Freeland *et al.* 2013; Ciotir *et al.* 2017). For example, *T. angustifolia* in North America is thought to have arrived from Europe several centuries ago (Ciotir *et al.* 2013), and ancestry assessments could test the hypothesis that historical interspecific hybridization led to genetic introgression into some *T. angustifolia* populations, which may help to explain regional erosion of species barriers. Locus-specific ancestry estimation analyses, markers, and the identification of potentially adaptive genes can all now be used to find evidence of ancient hybridization in *T. angustifolia* (Goulet *et al.* 2017; Taylor and Larson 2019). This high-quality draft genome and its comparisons with Poales species will be an indispensable resource for ongoing research into *Typha*, a genus that both sustains and threatens wetlands around the world.

## Data availability

Raw sequencing data can be found in the SR Archive (SRA) under accession number PRJNA751759. Genome assembly has been submitted to GenBank (JAIOKV000000000). Code used to generate the data can be found at https://gitlab.com/WiDGeT_TrentU/undergrad-theses/-/tree/master/Widanagama_2021. The *T. angustifolia* RNSseq data are in the SRA under SRR15541138. The *T. latifolia* transcriptome used can be found at https://figshare.com/articles/dataset/Typha_latifolia_leaf_transcriptome/5661727/1.

Supplementary material is available at *G3* online.

## Supplementary Material

jkab401_Supplementary_Data
