# Supplementary material for: Genome assembly, annotation, and comparative analysis of the cattail Typha latifolia
Source: G3 (Bethesda). 2021 Nov 22;12(2):jkab401. doi: 10.1093/g3journal/jkab401 (PMC9210280; doi:10.1093/g3journal/jkab401)
Supplement: jkab401_Supplementary_Data [file jkab401_supplementary_data.docx]

SUPPLEMENTARY FIGURES AND TABLES

**Table S1.** Chloroplast and mitochondrial genomes used to identify cpDNA and mtDNA in the reference genome using NUCmer.

| Species | Type | Accession |
| --- | --- | --- |
| *Typha latifolia* | chloroplast | NC_013823.1 |
| *Oryza sativa* | mitochondrion | NC_011033.1 |
| *Zea mays* | mitochondrion | NC_007982.1 |

**Table S2.** Sequencing read data, with coverage based on the genome size as estimated by k-mer frequency method (Figure S1).

| Library type | Sequencer | Coverage | Insert length (bp) | Sequences | Bases sequenced (bp) |
| --- | --- | --- | --- | --- | --- |
| Paired-end | Illumina HiSeqX | 545.55 | 151 | 458,843,049 | 138,570,600,798 |
| PacBio | PacBio Sequel II | 337.35 | 11,978 | 7,244,218 | 86,772,692,047 |

**Table S3.** Genome assembly statistics of *T. latifolia* using different short read assemblers. Unless specified otherwise, reads were trimmed using Trimmomatic. For more information on parameters used see Materials and Methods.

|  | Platanus | Platanus w/ Platanus_trim | ABySS | ABYSS, 20% of reads | SOAPdenovo2 |
| --- | --- | --- | --- | --- | --- |
| Genome Size (Mb) | 275.42 | 269.89 | 263.74 | 251.91 | 1,154.62 |
| Contigs | 533,899 | 497,397 | 365,565 | 259,147 | 29,219,132 |
| Contig N50 | 1.89 Kb | 1.87 Kb | 11.43 Kb | 22.59 Kb | 45 bp |
| Max sequence length (Kb) | 97.34 | 73.29 | 154.73 | 662.61 | 746 |
| Contigs > 50 Kb | 16 | 10 | 362 | 901 | 0 |
| % of contigs > 50 Kb | 0.35 | 0.22 | 9.46 | 32.58 | 0 |

**Table S4.** Genome assembly statistics of *T. latifolia* using DBG2OLC and Canu assemblers. DBG2OLC 1 used ABySS contigs made from all the Illumina reads. DBG2OLC 2 used ABySS contigs made from 20% of the Illumina reads.

|  | DBG2OLC 1 | DBG2OLC 2 | Canu |
| --- | --- | --- | --- |
| Genome size (Mb) | 193.16 | 179.77 | 287.63 |
| Contigs | 1,840 | 2,664 | 1,190 |
| Contig N50 | 132.07 Kb | 77.98 Kb | 8.71 Mb |
| Max sequence length | 934.40 Kb | 505.97 Kb | 18.70 Mb |
| Contigs > 10 Kb | 1,833 | 2,641 | 1,140 |
| Contigs > 25 Kb | 1,785 | 2,438 | 1,127 |
| Contigs > 50 Kb | 1,445 | 1,493 | 821 |
| % of contigs > 50 Kb | 92.34 | 77.35 | 95.54 |

**Table S5.** Nucleotide sequences aligned to organelle genomes.

| Scaffold Name | Scaffold Length (bp) | Scaffold Length (bp) – hardmasked | Type |
| --- | --- | --- | --- |
| tig00001508 | 440,773 | 393,189 | mitochondrion |
| tig00001509 | 224,853 | 109,004 | chloroplast |
| tig00001510 | 63,630 | 28,080 | chloroplast |

**Table S6.** Accession numbers to annotated genomes of species that the counts of different ncRNA types found in *T. latifolia*’s annotation were compared with.

| Species | Assembly Accession |
| --- | --- |
| *Ananas comosus* | GCF_001540865.1 |
| *Oryza sativa japonica* | GCF_001433935.1 |
| *Brachpodium distachyon* | GCF_000005505.3 |


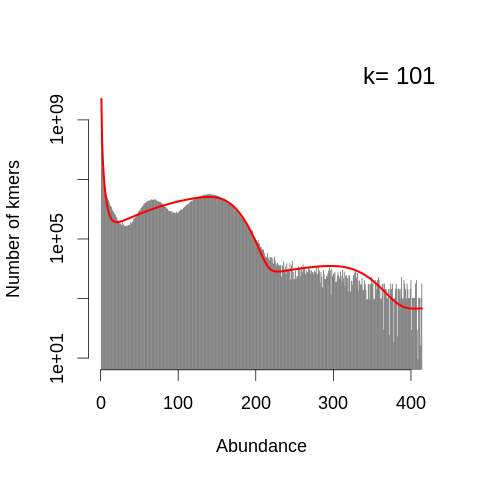


**Figure S1.** Estimation of *T. latifolia* genome size using k-mer frequency method.

**Figure S2.** MUMMER alignment of our *Typha latifolia* assembly to another recently released hybrid assembly (JAAWWQ010000000).

**Figure S3.** Mapping success (bwa mem) of unrelated re-sequenced *Typha* spp. samples aligned to our assembly and another hybrid assembly (JAAWWQ010000000).
